# Capsaicin Decreases Kidney Iron Deposits and Increases Hepcidin Levels in Diabetic Rats with Iron Overload: A Preliminary Study

**DOI:** 10.3390/molecules27227764

**Published:** 2022-11-11

**Authors:** Marisa López, Laura Quintero-Macías, Miguel Huerta, Alejandrina Rodríguez-Hernández, Valery Melnikov, Yolitzy Cárdenas, Jaime Alberto Bricio-Barrios, Enrique Sánchez-Pastor, Armando Gamboa-Domínguez, Caridad Leal, Xóchitl Trujillo, Mónica Ríos-Silva

**Affiliations:** 1 University Center of Biomedical Research, Universidad de Colima, Av. 25 de Julio #965, Col. Villas San Sebastian, Colima 28045, Mexico; 2 Faculty of Medicine, Universidad de Colima, Av. Universidad #333, Col. Las Víboras, Colima 28040, Mexico; 3 Belisario Domínguez Sección XVI, Pathology Department, Instituto Nacional de Ciencias Médicas y Nutrición Salvador Zubirán, Mexico City 14080, Mexico; 4 Centro de Investigaciones Biomédicas de Occidente, Instituto Mexicano del Seguro Social, Sierra Mojada No. 800, Col. Independencia, Guadalajara 44340, Mexico; 5 University Center of Biomedical Research, CONACyT-Universidad de Colima, Av. 25 de Julio #965, Col. Villas San Sebastian, Colima 28045, Mexico

**Keywords:** iron overload, capsaicin, diabetes mellitus, kidney, biomarkers

## Abstract

Iron overload (IOL) increases the risk of diabetes mellitus (DM). Capsaicin (CAP), an agonist of transient receptor potential vanilloid-1 (TRPV1), reduces the effects of IOL. We evaluated the effects of chronic CAP administration on hepcidin expression, kidney iron deposits, and urinary biomarkers in a male Wistar rat model with IOL and DM (DM-IOL). IOL was induced with oral administration of iron for 12 weeks and DM was induced with streptozotocin. Four groups were studied: Healthy, DM, DM-IOL, and DM-IOL + CAP (1 mg·kg^−1^·day^−1^ for 12 weeks). Iron deposits were visualized with Perls tissue staining and a colorimetric assay. Serum hepcidin levels were measured with an enzyme-linked immunosorbent assay. Kidney biomarkers were assayed in 24 h urine samples. In the DM-IOL + CAP group, the total area of iron deposits and the total iron content in kidneys were smaller than those observed in both untreated DM groups. CAP administration significantly increased hepcidin levels in the DM-IOL group. Urinary levels of albumin, cystatin C, and beta-2-microglobulin were similar in all three experimental groups. In conclusion, we showed that in a DM-IOL animal model, CAP reduced renal iron deposits and increased the level of circulating hepcidin.

## 1. Introduction

Iron overload (IOL) is common in type 2 diabetes (DM) [1], but the underlying mechanisms have not been completely elucidated. The relationship between IOL and DM appears to be bidirectional, based on reports that have shown that complications secondary to DM seem to be related to IOL. Both iron deficiency and IOL have been associated with impaired glucose tolerance in DM [2]; thus, it is not uncommon for DM and IOL to coexist in the same person.

Iron plays an important physiological role in various metabolic pathways. IOL disrupts iron homeostasis, which induces defective signaling. Thus, IOL causes alterations in metabolic activity through multiple mechanisms that contribute to DM, such as gluconeogenesis, lipolysis, and processes that determine fuel availability. These alterations affect the pancreas, liver, adipocytes, and muscle, where they lead to reduced insulin secretion, insulin resistance, and increased hepatic gluconeogenesis. The precise mechanisms that determine how these metabolic pathways are altered are diverse, and to date, they have not been fully clarified. However, ferroptosis seems to play a leading role in IOL [2,3]. Furthermore, IOL can increase oxidative stress, which contributes to complications including diabetic kidney disease (DKD) [4].

DKD is the primary cause of renal insufficiency worldwide [5]. Its management is focused on controlling glucose levels and associated risk factors. Blocking the renin–angiotensin system has been shown to be effective in slowing DKD progression [5], but the prevalence of DKD continues to be high. IOL is thought to contribute to kidney damage in DKD [6].

In the kidney, IOL increases filtration and iron capture in tubule cells, but high amounts of iron exceed the cells’ abilities to export and store iron as ferritin. This condition compromises the tubules’ ability to offset the toxic effects and tubule damage caused by iron [4]. Increased iron deposits have been observed in the kidneys of diabetic male rats with IOL [7], and kidney ferritin is increased in rats with IOL and DKD compared to controls [6]. Conversely, iron restriction improves kidney mitochondrial dysfunction and oxidative stress in diabetic rats [8]. Hepcidin, a hormone that regulates iron transport, plays a key role in controlling systemic iron homeostasis, but its levels are altered in patients with DM [9].

Capsaicin (CAP) is the main pungent compound of chili peppers. When bound to its receptor—transient receptor potential vanilloid 1 (TRPV1)—CAP can affect carbohydrate metabolism, depending on the timing and dose [10,11]. TRPV1 is reported to be expressed in the renal cortex and medulla [12,13]. The activation of TRPV1 in kidney tissues has been associated with a nephroprotective effect against fibrosis [14] because it diminishes inflammation and oxidative stress [15]. In addition, CAP acts as an antioxidant by reducing serum malondialdehyde levels [16]. It also reduces kidney disease biomarker levels, including albumin, C cystatin C, β2 microglobulin (β2M), and epidermal growth factor (EGF) [17], and it reduces the effects of IOL on hemoglobin levels [18] in diabetic rats. It has also been shown that the administration of CAP reduces lipid peroxidation (one of the main characteristics of ferroptosis) caused by IOL in the liver. Moreover, CAP exhibits iron-binding properties in brain tissues [19]. In the present study, we aimed to determine whether CAP reduces the effects of IOL in an experimental model of DM. To that end, we investigated changes in kidney iron deposits in male Wistar rats with experimental DM and IOL (DM-IOL), after chronic administration of CAP (1 mg·kg body weight^−1^·day^−1^) for 12 weeks.

## 2. Results

**Figure 1:**
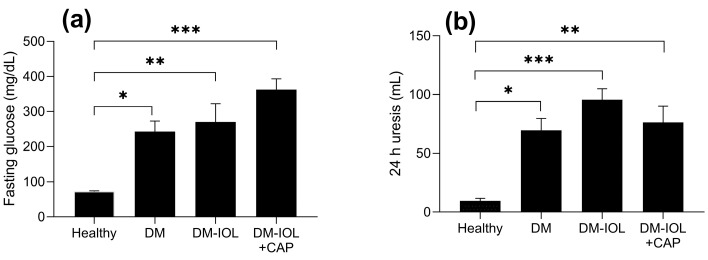
Effects of capsaicin (CAP) on (**a**) glucose and (**b**) uresis in diabetic (DM) rats with iron overload (IOL). There was no statistical difference (*p* > 0.05) between the DM groups. Data are expressed as the mean ± standard error. ANOVA and post-hoc Bonferroni test. DM: DM without IOL; DMIOL: DM and 12 weeks IOL; DM-IOL + CAP: DM and 12 weeks IOL + CAP. * *p* ≤ 0.05, ** *p* ≤ 0.01, *** *p* ≤ 0.001.

We explored the effect of CAP on kidney iron deposits by isolating and sectioning kidneys from diabetic (DM) or healthy rats. The kidneys were analyzed after 12 weeks in healthy untreated rats, after 12 weeks of IOL treatment in DM rats (DM-IOL), or after 12 weeks of CAP treatment in DM-IOL rats. Kidney tissue sections were histologically stained with Perls’ Prussian blue and analyzed with a colorimetric assay. Table 1 shows the morphological characteristics of the experimental groups. Body weights were reduced by CAP treatment in DM-IOL rats. Glucose levels and uresis were not significantly different between the DM and DM-IOL groups (Figure 1).

**Figure 2:**
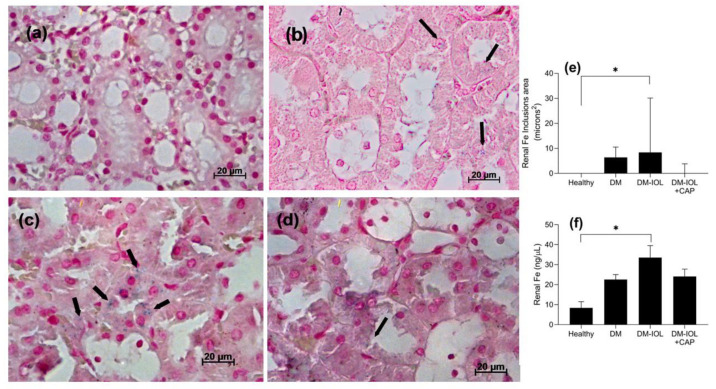
Representative images show that 12-week treatment with capsaicin (CAP, 1 mg·kg^−1^·day^−1^) reduced iron deposits observed in the kidney tissues of rat groups with iron overload (IOL). (*Top row*) Iron deposits detected with stain (arrows) in kidney tissues from (**a**) healthy rats; (**b**) in rats with diabetes (DM) only; (**c**) in DM with IOL (DM-IOL); and (**d**) in DM with IOL + CAP (DM-IOL + CAP); (**e**) Areas of iron deposits measured in kidneys from each group with Perls’ Prussian blue staining; (**f**) Total iron content in kidneys. Images were acquired at 40× with Axio Vision v.4.8. Scale bars: 20 µm. Graphs show the mean ± standard error. * *p* < 0.05, ANOVA and post-hoc Bonferroni test.

Iron deposits in the cytoplasm of tubule cells were stained blue, primarily in DM-IOL rats. In contrast, we did not find iron inclusions in healthy control rats. Moreover, iron deposits were not detected in the glomeruli or other nephron structures (Figure 2b–d). The CAP-treated group (DM-IOL + CAP) had significantly smaller areas of kidney iron deposits (Figure 2d,e, *p* < 0.05) and smaller glomerular diameters compared to the healthy and DM groups.

**Figure 3:**
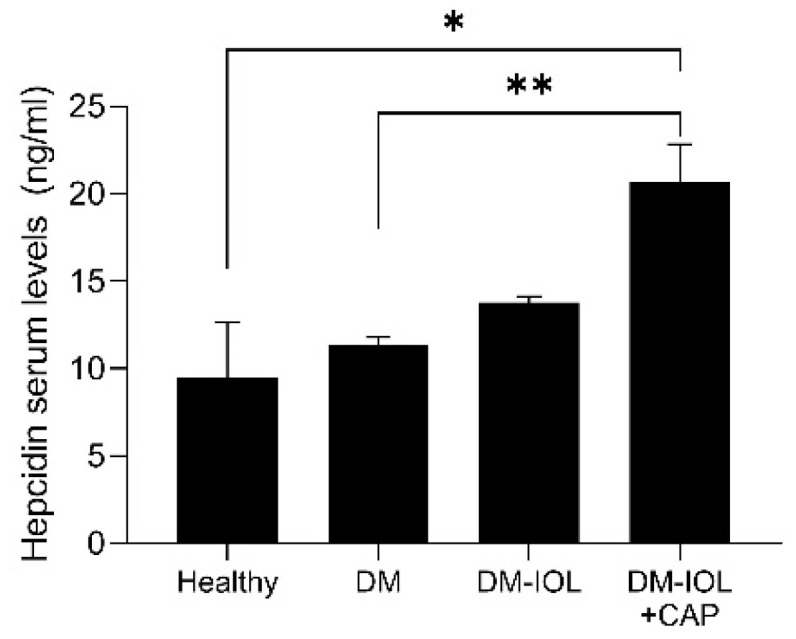
Serum hepcidin levels in healthy rats and in rats with diabetes (DM) and experimental iron overload (IOL), untreated or treated with capsaicin (CAP). Data are expressed as the mean ± standard error. * *p* < 0.05, ** *p* < 0.01, ANOVA and post-hoc Bonferroni test.

We observed that the levels of hepcidin were significantly higher in DM-IOL + CAP rats than in DM rats without IOL (Figure 3).

**Figure 4:**
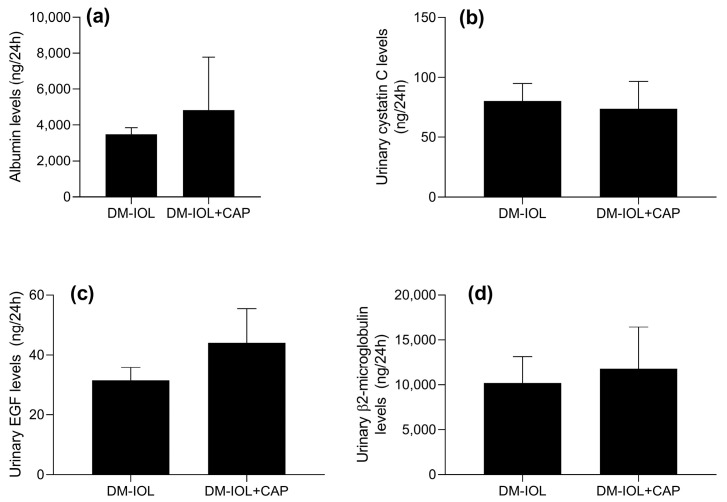
Capsaicin (CAP) effects on kidney disease biomarkers in rats with diabetes (DM) and iron overload (IOL), without or with capsaicin treatment (DM-IOL or DM-IOL + CAP, respectively). Graphs show changes in the levels of (**a**) albumin; (**b**) cystatin C; (**c**) EGF; and (**d**) beta-2-microglobulin in 24 h urine samples from experimental IOL groups. Urine samples were diluted 1:500. Data are expressed as the mean ± standard error. *p* > 0.05, Student’s t test with post-hoc Bonferroni test.

Next, we examined the effects of CAP on kidney disease biomarkers. Previous studies reported that CAP could modify these biomarkers in rats without IOL [17]. Here, we observed that, in rats with IOL (Figure 4a–d), CAP had no significant effects on albuminuria, cystatin C, β2M, or EGF levels.

## 3. Discussion

Current information is scarce about the effects of CAP in kidney tissues. In a previous study, we observed that CAP treatment affected some early kidney biomarkers and had a diuretic effect in both DM and healthy rats [17]. These findings suggested that CAP might interfere in the development of DKD. However, in that study, CAP was administered over an 8-week period and the rats did not have IOL. In contrast, here, we administered CAP for 12 weeks to DM rats after inducing IOL. Thus, study-design differences might explain the differences observed in uresis and glycemia between studies. Moreover, other studies showed that, after inducing diabetes, structural and functional changes in the kidney increased over time; thus, the effects of chronic CAP treatment may be diverse in different stages of DKD [20].

Our results showed that iron deposits declined in kidneys after chronic CAP administration in rats with DM and IOL. Previous studies in animal models with both DM and IOL showed that IOL increases kidney damage caused by DM [7]. Therefore, in the present study, the decline in iron deposits that we observed in rats treated with CAP suggested that CAP might interfere with the progression to diabetic nephropathy. However, we found that the levels of kidney-damage indicators in CAP-treated rats were similar to the levels observed in both DM rats and DM-IOL rats. Future studies might be able to identify the appropriate dose, timing, or stage for initiating CAP treatment to achieve an optimal effect on renal biomarkers. Nevertheless, although the kidney disease indicators were not lower in the CAP-treated group, that finding did not rule out the possibility that CAP could have provided a nephroprotective effect, due to the reduced quantity of iron deposits in the renal tubules.

In the kidney, iron regulation is mainly limited to the nephron tubule cells. The receptors and transporters related to iron homeostasis that have been identified in tubule cells are also expressed in other organs and tissues [4]. Iron regulatory mechanisms appear to be related to both systemic and local protection against iron toxicity or deficiency [4,21]. One of the main iron regulatory proteins is ferroportin. In kidney tubule cells, ferroportin has been identified at the basolateral membrane, which favors iron export to the blood. In contrast, the divalent metal transporter 1 (DMT1) has been identified in the apical membrane, which permits iron reabsorption through transepithelial iron transport [22,23]. In the kidneys of streptozotocin-induced (STZ) diabetic rats, DMT1 has been shown to be down-regulated and the transferrin receptor (TFr) up-regulated [24]. However, in genetically engineered diabetic (db/db) mice, DMTI and TFr protein expression levels are not different from those observed in wildtype controls, but ferroportin is up-regulated in renal cells [25]. Those findings suggested that kidney iron metabolism is managed differently in different DM models. Future experiments could evaluate the roles of different transporters in mechanisms involving iron regulation in different segments of the renal tubule. In particular, most iron reabsorption occurs in the convoluted proximal tubule, and it has been shown that DMT1, Zip8, and Zip14 transporters are not involved in this function [26].

Hepcidin, a hepatic hormone, induces the internalization of ferroportin and its lysosomal degradation [27]. As in DM models, in IOL models, increased hepcidin levels have been shown to inhibit cellular iron uptake. In our experiments, CAP appeared to potentiate this mechanism. It is well-known that the levels of iron in serum regulate hepcidin synthesis. This regulation acts through the BMP/SMAD signaling pathway, which up-regulates the transcription of hepcidin in response to increases in serum iron. Conversely, when serum iron levels are low, the BMP/SMAD pathway can be blocked by HIF and the iron response elements.

Other mechanisms for regulating iron homeostasis involve inflammatory proteins, erythroid regulators, and the more widely described pathway that activates IL-6 [28]. IL-6 pathway activation can induce hepcidin synthesis [29], and increments in IL-6 levels have been correlated with elevated glycemia in diabetes [30]. Our diabetic rats treated with CAP for 12 weeks exhibited elevated levels of glycemia (although the elevation was not significant). This increase in glycemia could have increased the levels of IL-6, and thus, induced hepcidin synthesis. Elucidation of the mechanism by which CAP increases hepcidin levels will require further research.

On the other hand, in the context of IOL, CAP administration could have effects independent of systemic regulation via hepcidin. For example, in IOL, intracellular excess of free labile iron (Fe^2+^) increases the production of free radicals by acting as catalyst in the Fenton reaction. This reaction contributes to severe lipid peroxidation, and then, ferroptosis [31]. Reportedly, CAP acts as an iron chelator in homogenized healthy rat brain tissues [32]. Moreover, in a model of ischemia-reperfusion, a CAP analog has been shown to inhibit intestinal ferroptosis by activating TRPV1 through an enhancement of glutathione peroxidase 4 activity [33]. Additional studies are needed to elucidate the effects of CAP on ferroptosis in the kidney.

In conclusion, we demonstrated that DM-IOL rats treated with CAP exhibited a significantly lower frequency of iron inclusions in kidney tissues and lower total iron content compared to rats with DM-IOL. Moreover, our results indicated that chronic CAP administration could increase the production of circulating hepcidin. These findings contributed to a better understanding of the role and mechanism of CAP in IOL-associated diabetic nephropathy. Our results may lead to new strategies for the early prevention and treatment of this complication in patients with diabetes.

## 4. Materials and Methods

### 4.1. Animals

This study included 15 2-month-old male Wistar rats (Envigo, Inc. Huntingdon Life Sciences Ltd., Cambridgeshire England, UK) that weighed 250 ± 50 g. All animals were maintained according to the specific protocols for laboratory animals at the animal facility of the Biomedical Research Center at the University of Colima (Universidad de Colima), México. Animals were housed in pathogen-free conditions, under 12 h light/12 h dark conditions at 22 ± 2 °C. Rats were fed with generic rodent feed (Envigo, Harlan Laboratories, Inc., Madison, WI, USA). During the experiment, food and water were supplied ad libitum, except during food deprivation periods before blood draws. Hemolyzed samples were discarded, and the rats that died during the study were not replaced as the number of deaths was lower than predicted.

All experimental protocols and animal management protocols were performed in accordance with the ethical standards and technical specifications for the care and use of laboratory animals. Rats were euthanized without pain or distress by administering increasing doses of anesthesia (intraperitoneal lethal doses of pentobarbital sodium, Pets pharm, Mexico). The Ethics Committee at the University of Colima approved all protocols (2016–05a). This study was carried out in compliance with the ARRIVE guidelines.

### 4.2. Experimental Protocol

The experimental period was 12 weeks. DM was induced in all rats and they were maintained without anti-diabetic treatment. DM rats were randomized into three groups, and IOL was induced in two groups. Thus, the groups included DM: rats with experimental diabetes, without iron overload, plus vehicle (mock treatment, *n* = 5); DM-IOL: rats with experimental diabetes, with iron overload, plus vehicle (*n* = 5); and DM-IOL + CAP: rats with experimental diabetes, with IOL, and with CAP treatment for 12 weeks (*n* = 5). One rat in the DM group died during the study. An additional group of healthy rats without IOL was included (*n* = 4).

To induce experimental diabetes, rats received a single intraperitoneal injection of 45 mg·(kg body weight)^−1^ STZ (Sigma-Aldrich Co., St. Louis, MO, USA). The diabetic state was confirmed when the fasting blood glucose measurement was ≥200 mg·dL^−1^ [18]. To induce IOL, we administered polymaltose iron (Takeda, Mexico, SA de CV, under license of Vifor International, Switzerland) by oral gavage at a dose of 3 mg·(kg body weight)^−1^·day^−1^, every day for 12 weeks. Prior to administration, iron polymaltose was diluted 1:50 [18]. The administration of polymaltose iron began 1 week after the induction of DM.

**Figure 5:**
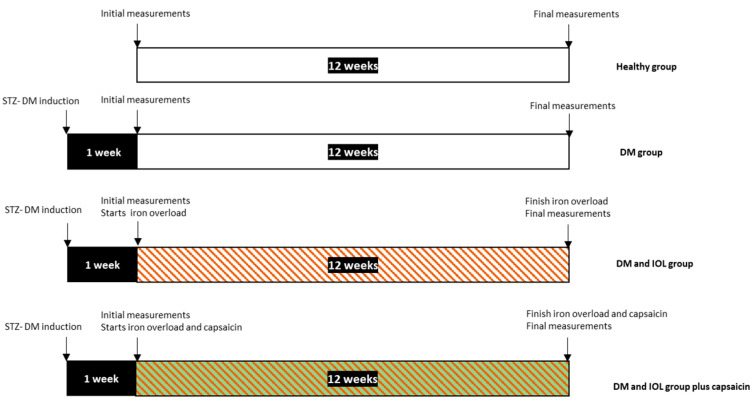
Timeline of the experimental groups.

Prior to administration, CAP (Sigma-Aldrich, St. Louis, MO, USA) was dissolved in 10% Tween 80 (Sigma-Aldrich, St. Louis, MO, USA) and 10% ethanol. We added a 0.9% saline solution to this mixture, at a ratio of 2:1:1. This CAP solution was administered subcutaneously, immediately after it was prepared, at a dose of 1 mg·(kg body weight)^−1^·day^−1^, in an injection volume of approximately 0.1 mL [16]. CAP was administered daily for 12 weeks, starting 1 week after the induction of diabetes. Thus, the IOL groups received the CAP or vehicle injection at the same time they received the polymaltose iron (Figure 5).

Blood glucose was measured after 12 h of fasting. Briefly, peripheral blood was drawn from the tip of the tail, and glucose was measured with an Accu-Chek Active autoanalyzer (Roche, Mannheim, Germany). For hepcidin assays, animals were anesthetized with pentobarbital and blood samples were collected with an intracardiac puncture. Samples were stored at −70 °C. Hepcidin levels were determined with an ELISA kit (MBS017183, Mybiosource, San Diego, CA, USA) [34].

### 4.3. Analysis of Iron Tissue Content

Kidneys were removed immediately after sacrifice, and tissues were fixed in 10% buffered formaldehyde. The right kidneys were embedded in paraffin and sectioned into 3 μm-thick slices. Some sections were mounted onto slides and stained with hematoxylin/eosin for morphological analysis. Other sections were stained with Perls Prussian blue to evaluate iron deposition. Left kidneys were frozen at −70° immediately after removal to determine the total iron content. The hypertrophy index was calculated as the average wet weight of both kidneys, compared to the total body weight as a ratio.

For every tissue section, the total-surface image was acquired with an automated Axio Imager microscope (Carl Zeiss, Inc., New York, NY, USA) equipped with an AxioCam Mr 5 camera (Carl Zeiss, Inc., New York, NY, USA). Sections were visualized in a bright field with an Axiofluor 40× lens. All images were semi-automatically processed with Axio Vision image analysis software (Carl Zeiss, Inc., New York, NY, USA) and macros that were specially designed to detect spots in the blue channel. For each blue spot, the surface area within the blue channel was measured. All photomicrographs were acquired under the same conditions of light and exposure. Analyses were completed by one pathologist blinded to the experiments. From each tissue sample, three to four sections were analyzed, and the mean was calculated for each rat. This semi-quantitative technique provided an adequate correlate to the content of iron in tissues [34,35]. Total iron content was determined in homogenized kidney tissue with a total iron assay (Mybiosource, Cat no. MBS2567950), according to the manufacturer’s instructions.

### 4.4. Measurements of Kidney Disease Biomarkers

Animals were maintained in metabolic cages for 24 h urine collections. After 24 h, the collected urine was centrifuged at 400× *g* for 5 min and then frozen at −80 °C until analysis. For the analyses, thawed samples were divided into aliquots, diluted 1:500, and placed into 96-well plates. The plates contained magnetic pearls of a Toxicity Multiplex Assay kit (MILLIPLEX MAP Rat Kidney Toxicity Magnetic Bead Panel 2, Cat. RKTX2MAG-37K, from EMD Millipore Co., Charles, MO, USA). Analyses were performed according to the manufacturer’s recommendations to determine the levels of albumin, cystatin C, β2M, and EGF. All biomarker concentrations were evaluated with a Magpix instrument (Luminex X-MAP, Vercelli, Italy).

### 4.5. Statistical Analysis

We performed descriptive statistical analyses. The variables are expressed as the mean ± standard error of the mean. *p*-values < 0.05 were considered statistically significant. Analyses were performed with SPSS v22 software. Comparisons were performed with Kruskal–Wallis or ANOVA tests and then the Bonferroni post-hoc analysis was applied.

## Figures and Tables

**Table 1:** Morphologic characteristics of diabetic rats (DM) with or without iron overload (IOL) and untreated or treated with capsaicin (CAP).

Characteristic	Healthy	DM	DM-IOL	DM-IOL + CAP
Initial body weight (g)	291.5 ± 1.9	288 ± 1.58	278.6 ± 6.8	271.2 ± 10.2
Final body weight (g)	455.6 ± 13.6 ^£^	370 ± 29.3 ^£^	281.1 ± 9.1	268.4 ± 9.8
Kidney weight (g)	1.19 ± 0.06	1.4 ± 0.09	1.3 ± 0.02	1.2 ± 0.07
Kidney hypertrophy index (mg/g)	2.6 ± 0.2 ^£^	4.1 ± 0.3	4.8 ± 2.2	4.6 ± 0.2
Glomerular diameter (μm)	211.8 ± 8.1	206.4 ± 14.6	221.4 ± 6.7	96.2 ± 1.4 ^£^

Values are expressed as the mean ± SEM. ^£^ *p* < 0.001 vs. all groups, ANOVA, post-hoc Bonferroni.

## Data Availability

The data presented in this study are openly available in https://doi.org/10.6084/m9.figshare.21534345.v1 (created on 10 November 2022).
